# Degree of Hybridization in Seed Stands of *Pinus engelmannii* Carr. In the Sierra Madre Occidental, Durango, Mexico

**DOI:** 10.1371/journal.pone.0152651

**Published:** 2016-04-11

**Authors:** Israel Jaime Ávila-Flores, José Ciro Hernández-Díaz, Maria Socorro González-Elizondo, José Ángel Prieto-Ruíz, Christian Wehenkel

**Affiliations:** 1 Doctorado Institucional en Ciencias Agropecuarias y Forestales, Universidad Juárez del Estado de Durango, Apdo, Postal 741, Zona Centro, Durango, Durango, México, C.P., 34000; 2 Instituto de Silvicultura e Industria de la Madera, Universidad Juárez del Estado de Durango, Apdo, Postal 741, Zona Centro, Durango, Durango, México, C.P., 34000; 3 CIIDIR Unidad Durango, Instituto Politécnico Nacional, Sigma 119 Fracc, 20 de Noviembre II, Durango, Durango, México, C.P., 34220; 4 Facultad de Ciencias Forestales, Universidad Juárez del Estado de Durango, Blv. Durango y Ave, Papaloapan s/n. Col. Valle del Sur, Durango, Durango, México, C.P., 34120; Aristotle University of Thessaloniki, GREECE

## Abstract

Hybridization is an important evolutionary force, because interspecific gene transfer can introduce more new genetic material than is directly generated by mutations. *Pinus engelmannii* Carr. is one of the nine most common pine species in the pine-oak forest ecoregion in the state of Durango, Mexico. This species is widely harvested for lumber and is also used in reforestation programmes. Interspecific hybrids between *P*.*engelmannii* and *Pinus arizonica* Engelm. have been detected by morphological analysis. The presence of hybrids in *P*. *engelmannii* seed stands may affect seed quality and reforestation success. Therefore, the goals of this research were to identify introgressive hybridization between *P*. *engelmannii* and other pine species in eight seed stands of this species in Durango, Mexico, and to examine how hybrid proportion is related to mean genetic dissimilarity between trees in these stands, using Amplified Fragment Length Polymorphism (AFLP) markers and morphological traits. Differences in the average current annual increment of putative hybrids and pure trees were also tested for statistical significance. Morphological and genetic analyses of 280 adult trees were carried out. Putative hybrids were found in all the seed stands studied. The hybrids did not differ from the pure trees in vigour or robustness. All stands with putative *P*. *engelmannii* hybrids detected by both AFLPs and morphological traits showed the highest average values of the Tanimoto distance, which indicates: i) more heterogeneous genetic material, ii) higher genetic variation and therefore iii) the higher evolutionary potential of these stands, and iv) that the morphological differentiation (hybrid/not hybrid) is strongly associated with the Tanimoto distance per stand. We conclude that natural pairwise hybrids are very common in the studied stands. Both morphological and molecular approaches are necessary to confirm the genetic identity of forest reproductive material.

## Introduction

Hybridization and backcrossing to one or both of the parental types can lead to incorporation of alleles from one taxon into the gene pool of the other [1]. Interactions between environment and genetic structure can then lead to segregation of a novel taxon from parental types. Depending on the degree of differentiation, hybrid offspring of different taxa may be identified as species, subspecies, variants or races [2] [3]. Hybridization is an important evolutionary force, because interspecific gene transfer can introduce more new genetic material than is directly generated by mutation [4]. At least 30%, and possibly as much as 80%, of all species may have been originated by hybridization [5].

Plant hybridization often leads to the formation of hybrid zones [6]. As narrow geographic regions and “tension zones”, hybrid zones are active sites of evolutionary change and high levels of genetic variation [1] [3]. Stable hybrid zones are not suitable for the process of hybrid speciation [7]. The great majority of hybrid zones are maintained in equilibrium between dispersal and selection and may remain in balance for long periods [8]. In contrast, a hybrid species probably originates from a hybrid founder event, in which one or more early generation hybrids populate a new area and therefore become spatially or ecologically isolated from the parent species [7] [9].

In general, hybrids are unfit relative to their ancestors, in particular because of postmating reproductive barriers. These common barriers comprise hybrid weakness or inviability, hybrid sterility and hybrid breakdown. However, the exception applies that first generation (*F*_*1*_) hybrids, particularly between geographic races or closely related species, tend to exceed their parents in vegetative vigour or robustness [10].

Extensive variation in viability and fertility is observed both within and between interspecific hybrid generations. Some genotypic classes of hybrids possess lower, equivalent or higher levels of fitness relative to their parental taxa. Levels of variability tend to be highest in the second generation (*F*_*2*_) and first back-cross (*BC*) generations. Successive hybrid generations are characterized by lower levels of variability and increased fecundity and viability as a result of natural (differential) selection [10] [11].

Although most studies concerning hybridization are based on morphological evidence, introgression or hybridization are not necessarily indicated by the phenotypic expression of characters of one taxon in another. Identical characters in species may occur as a result of phenotypic plasticity, convergent evolution, or simply common ancestry [12]. Furthermore, individuals from hybrid swarms that have obtained most of their genes from one of the parental taxa are often morphologically indistinguishable from that taxon [13].

The use of molecular markers has shown that interspecific hybridization is even more common than assessed by morphological and cytogenetic evidence alone. The hybrid nature of many species has indeed been confirmed by molecular studies [14] [15] [16]. Some pronounced advantages of molecular markers over morphological markers are: i) absence of pleiotropic effects [17], ii) lack of deleterious or strong epistatic effects, iii) a high number of molecular markers can usually be identified, iv) most are multilocus, allowing many markers to be considered simultaneously, v) numerous alleles, per locus, may exist for some marker types [18], and that vi) genotypes can be identified at either the whole plant, tissue, or cellular levels [19]. Amongst other molecular markers, Amplified Fragment Length Polymorphism (AFLP) markers have been used effectively to detect both interspecific hybridization and introgression in animals, fungi and plants [20] [21] [22] [23] [24] [25].

Hybridization has been reported for very few Mexican pine species (e.g. [26] [27] [28] [29]). Recent diversification followed by secondary contact and hybridization may explain complex structures of intra- and interspecific morphological and genetic variation in the approximately 49 North American hard pines (*Pinus* section *Trifoliae* with subsections *Australes*, *Contortae*, and *Ponderosae*) [30]. *Pinus* subsection *Ponderosae* comprises approximately 17 species distributed from western Canada to Nicaragua. Although members of this group are of great ecological and economic importance, phylogenetic relationships between species are poorly understood [31] [32] [33]. Because of the very strong phylogenetic relationships among these species [34] [35] [30] and their very weak reproductive barriers, hybridization between *Ponderosae* species is quite possible [36][37][38][39]. Species divergence is recent and hybrid ancestry in some *Ponderosae* subsection species is inferred from an incomplete lineage sorting [40].

Apache pine (*P*. *engelmannii* Carr.) is, along with *Pinus arizonica* Engelm., *Pinus arizonica* var. *cooperi* (C.E. Blanco) Farjon and *Pinus durangensis* Martínez, one of the most commonly occurring *Ponderosae* species in the Mexican Sierra Madre Occidental [41] [31] and has a limited distribution in the mountains of Arizona and New Mexico. Interspecific hybrids between Apache pine, interior ponderosa pine (*P*. *ponderosa* var. *scopulorum*) and Arizona pine (*P*. *arizonica*) have been detected by morphological analysis [42] [43]. The presence of hybrids in seed stands may affect seed quality and reforestation success [9] [10] [44].

*Pinus engelmannii* grows on dry to moderately moist canyon slopes, ridges, mesas, lower slopes, valleys and streamside terraces, at elevations of 1,500–2,700 m, in climates ranging from semiarid with bimodal precipitation to temperate-subhumid with most precipitation falling in summer [45] [46]. It is one of the nine most frequent pine species in the Sierra Madre Occidental in the state of Durango, Mexico [47]. It is widely harvested for lumber and is also used in reforestation programs in Durango [48].

The goals of this research were to identify introgressive hybridization between *P*. *engelmannii* and other pine species in eight seed stands of this species in the state of Durango, Mexico, and to examine how hybrid proportion is related to mean genetic dissimilarity between trees in these stands, using Amplified Fragment Length Polymorphism (AFLP) markers and morphological traits. We also hypothesized no differences in the average current annual increment of putative hybrids and pure trees, given that all the studied trees were healthy, dominant and superior phenotypes (plus trees).

## Materials and Methods

We confirm that we provide the specific location of the field studies (Table 1). None vertebrate study was carried out. We confirm that the owners of the lands gave permission to conduct these studies on those sites.

**Table 1:** Details of the studied stands of *Pinus engelmannii*.

Stand	No.	Code	Location	Property	Municipality	Latitude (N)	Longitude (W)	Elevation(m)	Age (years)
W	1	LM	La Mesa	Ejido Pueblo Nuevo	Pueblo Nuevo	23° 26' 14.0"	105° 03' 11.4"	2,327	51–86
W	2	CU	Cumbres	Predio Particular Cumbres	Durango	23° 51' 55.3"	105° 14' 27.3"	2,441	38–103
W	3	ARC	Adolfo Ruiz Cortinez	Ejido Adolfo Ruiz Cortinez	Durango	23° 42' 46.7"	105° 17' 13.7"	2,330	48–87
W	4	NP	Nueva Patria	Ejido Nueva Patria	San Dimas	24° 03' 06.8"	105° 29' 21.2"	2,268	32–86
E	5	RC	Rancho Los Castro	Ejido La Casita	Durango	23° 44' 38.3"	104° 45' 29.5"	2,302	22–69
E	6	R	El Río	Ejido La Casita	Durango	23° 43' 26.8"	104° 47' 27.1"	2,335	22–55
E	7	MC	Mesa Cebollas	Comunidad San Bernardino de Milpillas	Pueblo Nuevo	23° 21' 28.6"	104° 50' 27.2"	2,543	51–86
E	8	MCO	Mesa Coyotes	Comunidad San Bernardino de Milpillas	Pueblo Nuevo	23° 25' 46.9"	105° 01' 20.3"	2,315	22–50

W = Western, E = Eastern.

### Sampling sites

**Fig 1:**
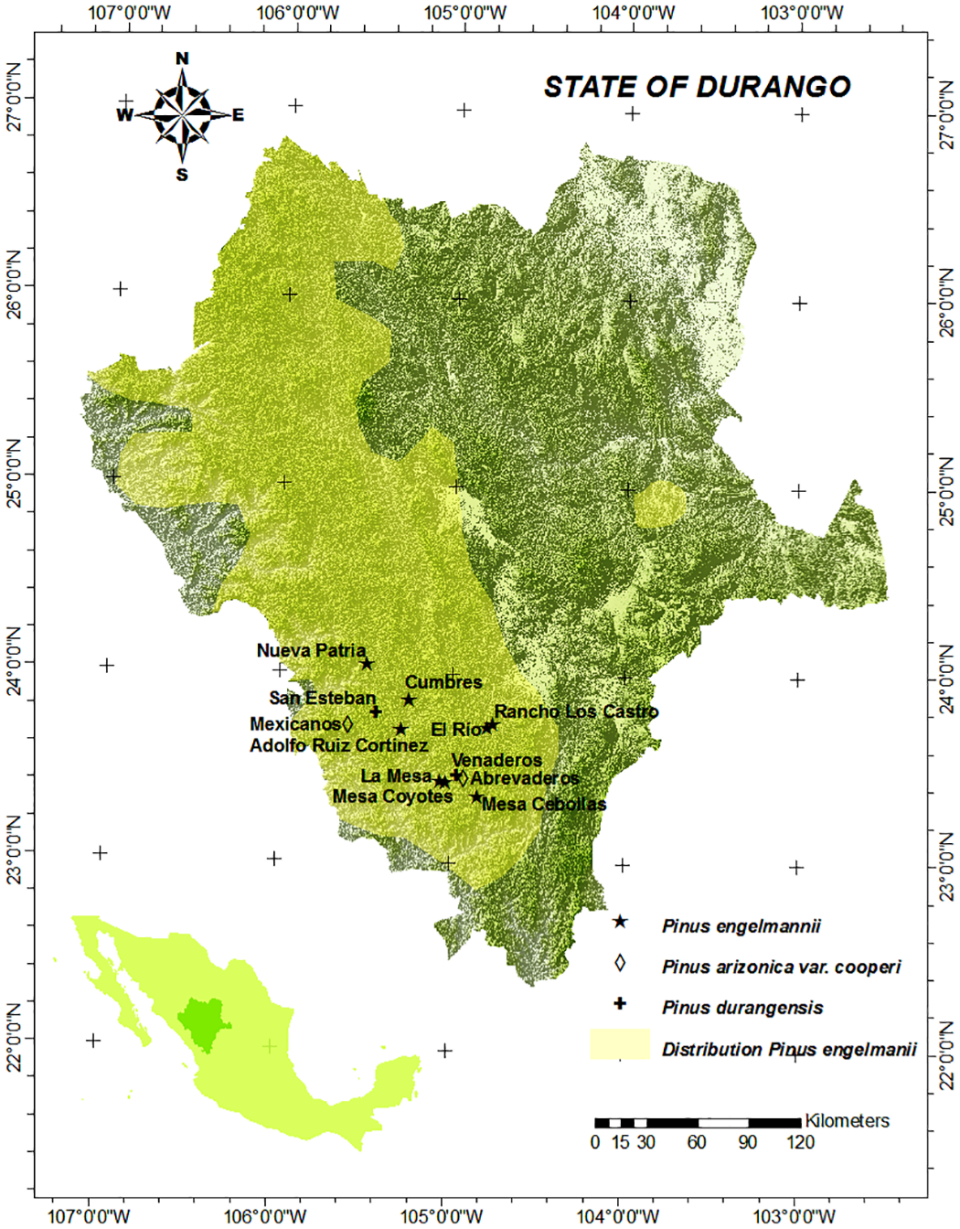
Location of the *Pinus engelmannii*, *P*. *arizonica* var *cooperi*, and *P*. *durangensis* stands. Location of the *Pinus engelmannii*, *P*. *arizonica* var *cooperi*, and *P*. *durangensis* stands in the state of Durango (NW Mexico). The distribution of *P*. *engelmannii* was taken from Little [78]. Data sources: Own compilation based on freely-accessible digital maps from INEGI, Mexico (http://www.inegi.org.mx/geo/contenidos/mapadigital/). The shp format of the distribution of *P*. *engelmannii* is freely available at: http://esp.cr.usgs.gov/data/little/pinuenge.pdf (accessed July 2015).

Eight *Pinus engelmannii* (*Pe*) seed stands in the state of Durango (NW Mexico) were analyzed: 1) La Mesa (LM), 2) Cumbres (CU), 3) Adolfo Ruiz Cortínez (ARC), 4) Nueva Patria (NP), 5) Rancho Los Castro (RC), 6) El Río (R), 7) Mesa Cebollas (MC), and 8) Mesa Coyotes (MCO). These uneven-aged seed stands are located in natural populations (Table 1 and Fig 1).

For analysis of the genetic structure, needles were sampled from 280 adult, dominant and superior phenotypes (plus trees) of *Pinus engelmannii* (35 individuals per stand), in 2013 (for selection criteria, see Wehenkel et al. [49]). The spacing was similar for all the sampling trees. The mean distance between these trees was 39 m. For purposes of comparison, further investigations were carried out on 70 adult trees of *Pinus arizonica* var. *cooperi* (*Pco*) from two seed stands (Abrevaderos (AB) and Mexicanos (MEX)) and 70 adult trees of *Pinus durangensis* (*Pdu*) from another two seed stands (Venaderos (VEN) and San Esteban (SE)), close to the *P*. *engelmannii* stands under study (Fig 1).

Morphological analysis of all trees was conducted in the field. Samples of cones and branchlets were collected for taxonomic determination in the laboratory, which was based on morphological characters from needles, branchlets and cones, supported with field data of the bark texture and branching pattern of the trees, comparing with descriptions of the species in [48] and [50]. Vouchers were deposited in the herbarium of the Centro Interdisciplinario de Investigación para el Desarrollo Integral Regional (CIIDIR) at Durango. Dasometric variables were recorded for each *P*. *engelmannii* tree, including age, diameter at breast height (DBH) and average current annual increment (CAI) at age 20 years, because the tree growth depends on the tree age (Table 1).

### AFLP analysis

AFLP fingerprints were established according to the protocol described by Vos et al. [51]. DNA was extracted employing the DNeasy96 plant kit (QIAGEN) and digested with restriction enzymes EcoRI and MseI. Double-stranded MseI and EcoRI adaptors were ligated to the ends of the restriction fragments to generate template DNA. The restriction/ligation reaction was carried out at room temperature overnight. Subsequently, pre-selective amplification was conducted using the diluted ligation mix and primer combination E01/M03 (EcoRI-A/MseI-G). The final amplification reaction was initiated at 72°C for 2 min, followed by 20 cycles each consisting of 94°C for 10 sec, 56°C for 30 sec, and 72°C for 2 min, and a final step at 60°C for 30 min.

Selective amplification was carried out with the fluorescent-labelled (FAM) primer pair E35 (EcoRI-ACA) and M63+C (MseI-GAAC) for *P*. *engelmannii*, *P*. *durangensis* and *P*. *arizonica* var. *cooperi*. The fourth selective base was added to reduce the total number of peaks. Selective PCR cycling started at 94°C for 2 min, followed by 10 cycles, each consisting of 10 sec at 94°C, 30 sec at 65°C and 2 min at 72°C. The 65°C annealing temperature of the first cycle was subsequently reduced by 1°C for the next 10 cycles and continued at 56°C for 30 sec for the remaining 23 cycles, and was completed with a final extension step at 60°C for 30 min. All PCR reactions were carried out in a Peltier Thermal Cycler (MJ Research,Waltham, Massachusetts). The amplified restriction products were electrophoretically separated in a Genetic Analyzer (ABI 3100 16 capillaries), along with the internal size standard GeneScan 500 ROX (fluorescent dye ROX) from Applied Biosystems, Foster City, California, USA. The size of the AFLP fragments was resolved with the GeneScan 3.7 and Genotyper 3.7 software packages (Applied Biosystems) [52] [53] [54].

Scoring was fully automated and only strong and high quality fragments were considered. Only fragments above the signal threshold of 50 (minimum peak height) (according to ABI manual) and with a maximum peak width of 1.0, and fragment size ranging between 75 to 450 bp were considered. Two fragments were recorded only when the peak-peak distance between two signals was at least 0.5 bp [53].

Quality and reproducibility were tested by including reference samples in each plate and independent repetition (replicate PCRs) of at least 16 samples (i.e. a minimum of 16 individuals randomly chosen from each plate). All replicates presented the same AFLP pattern as in the first analyses (for further details, see [53]).

Finally, three binary AFLP matrices were created from the presence (code 1) or absence (code 0) at potential band positions. Each band detected corresponded to the presence of a dominant genetic variant (plus phenotype) with unknown mode of inheritance of this potential band position (detected fragment length) [54] [55]. The absence of a band reflected the presence of only recessive genetic (allelic) variants at the given position (locus).

Because *Pe*, *Pco* and *Pdu* are very closely related taxa [30] [34] [35] [40] and their physical and reproductive barriers are very weak [36] [37] [38], the degree of homoplasy was not expected to significantly increase [56] [57] [58].

To minimize the impact of size homoplasy [59] [60] and technical artifacts [55], only polymorphic loci with presence frequency between 5 and 95% (based across all 280 genotyped individuals) were selected for further study [61].

### Hybrid identification

If a *P*. *engelmannii* stand includes hybrid trees, these should possess a genome that is a blend of alleles derived from both *P*. *engelmannii* and other pine species, caused by gene flow. These hybrids are detectable by genetic and/or stable morphological traits (whose expression is under exclusive genetic control), as stated in [62] and [63] regarding natural hybridization within seed sources and hybridization in sympatric populations of *Pinus echinata* Mill. and *Pinus taeda* L., respectively.

In order to test the hypothesis that genetic introgression exists between *P*. *engelmannii* and other pine species, 280 *P*. *engelmannii* trees were screened. The alternative hypothesis was that there is no hybridization among the studied species.

In order to determine the degree of hybridism, 204 AFLP markers were used in four separate STRUCTURE (version 2.3.4) analyses [64] [65] to compare i) *P*. *engelmannii* to *P*. *arizonica* var. *cooperi*, ii) *P*. *engelmannii* to *P*. *durangensis* separately for the western and the eastern stands.

The Bayesian clustering method, as implemented in STRUCTURE was used to test whether affiliation of individuals to species on the basis of morphology was congruent with the assignment based on the AFLP markers. The population number (*K*) = 2 was used for this purpose. If the probability of *P*. *engelmannii* affiliation of a putative *P*. *engelmannii* tree was less than 95% according to STRUCTURE, then that individual was recorded as a candidate hybrid. The affiliation probability was measured by the proportion of the dominant STRUCTURE populations in the eight *P*. *engelmannii* studied stands. Individuals were identified as first-generation (*F*_*1*_) hybrids when the probability of *P*. *engelmannii* affiliation of a putative *P*. *engelmannii* tree was in the range 45–55%.

Then, in order to detect the degree of hybridization by morphological traits, vegetative and reproductive characters were assessed by using 14 qualitative and quantitative traits. Any tree displaying differences in these traits according to [48] and [50] was considered to be a putative hybrid. Character traits for morphologically "pure" *P*. *engelmannii* are shown in parenthesis: crown apex (rounded), crown density (open), twig diameter (1–3 cm), needle length (20–43 cm), needle width (1–2 mm), needle thickness (ca 1 mm), needle colour (dull pale green), number of stomata on dorsal face of needle (6–23), needle position (spreading-ascending to slightly drooping), young sheath length (2–4 cm); mature cone shape (ovoid to oblong), mature cone length (7–16 cm), mature cone width (6–12 cm), cone apophysis (elongate, strongly raised toward cone base). Characters not useful for distinguishing *P*. *engelmannii* from other related species (e.g. number of needles per fascicle) were not considered in this study.

### Association between genetic distance (*TD*) and hybrid frequency per stand

To test the relationship between genetic distance and hybrid frequency (*f*_*hyp*_) in each *P*. *engelmannii* stand, the AFLP data were also used to calculate the mean genetic dissimilarity between the binary vectors of two individuals in each stand, *a* and *b*, using the Tanimoto distance (*TD*) [66]. The relationship between mean *TD*_*ab*_ and hybrid proportion per stand was then computed by the covariation (*C*) described by Gregorius et al. [67]. This method can detect types of covariation that are monotonous but not necessarily linear. *C* varies from -1 to 1, where *C* = 1 shows an entirely positive covariation and *C* = -1 a strictly negative covariation. If the denominator is zero, *C* is indeterminate [67]. In order to test the possibility that the observed degrees of covariation *C*[mean *TD*_*ab*_ x *f*_*hyp*_] were only produced by random events, rather than directed forces, a one-sided permutation test was performed (here 10,000 permutations; *P*(*Z ≥ C*) < 0.05) [68].

### Differences in average current annual increment of putative hybrids and pure *P*. *engelmannii* trees

A permutation test based on randomly chosen reassignments was used to test whether the observed differences (*Diff*) in the mean values of the average current annual increment (CAI) of DBH at age 20 years of putative hybrids and pure trees in each *P*. *engelmannii* stand occur as random events, rather than by directed forces. If the *P*(*Z* ≥ *Diff*) is smaller than 0.05, we can expect statistically significant differences [68]. The average current annual increment (CAI) of DBH at age 20 years was determined from cores of wood taken with a Pressler borer.

## Results

The AFLP primer combination resulted in 204 polymorphic bands of 75–450 base pairs across all individuals of *Pinus engelmannii* (*Pe*), *P*. *arizonica* var. *cooperi* (*Pco*) and *P*. *durangensis* (*Pdu*). *Pe* shared 87% of AFLP fragments with *Pco* and 90% with *Pdu*.

**Fig 2:**
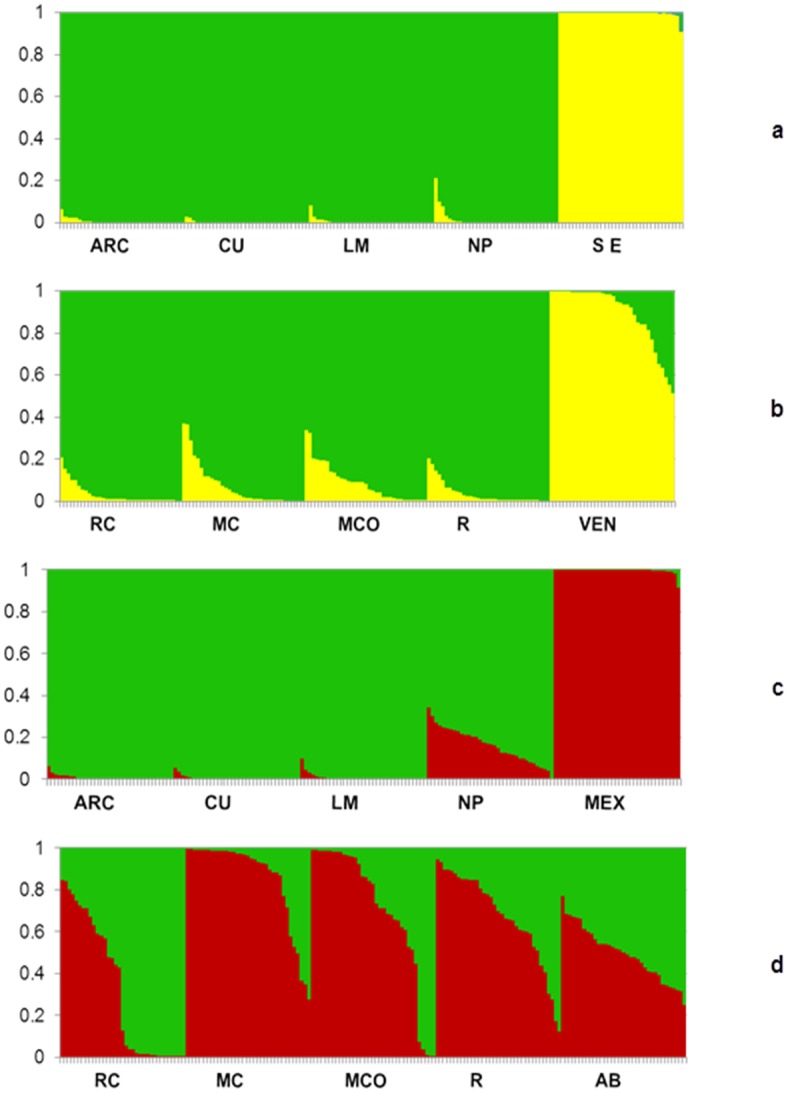
STRUCTURE results simulated with K = 2. STRUCTURE results for the eight *Pinus engelmannii* stands (RC: Los Castro, MC: Mesa Cebollas, MCO: Mesa Coyotes, R: El Rio, ARC: Adolfo Ruiz Cortinez, CU: Cumbres, LM: La Mesa, NP: Nueva Patria), two *Pinus durangensis* stands (**a)** SE: San Esteban; (**b)** VEN: Venaderos, and two *Pinus arizonica* var. *cooperi* stands (**c)** MEX: Mexicanos, (**d)** AB: Abrevaderos), Durango, Mexico. Data were simulated with K = 2, as described in Material and Methods.

Fig 2 illustrates the STRUCTURE results for the eight *Pe* stands, two *Pco* stands and two *Pdu* stands, simulated with the population number (*K*) = 2. *Pe* individuals are those most clearly separated from the *Pdu* and *Pco* individuals.

According to the results of STRUCTURE analyses, hybrids were found in all the studied stands. On the basis of morphological features, hybrids occurred in 37.5% of the stands. These hybrids were probably *Pe* x *P*. *arizonica* var. *arizonica* and in one case, *Pe* x *P*. *maximinoi*. Forty-two *Pe* x *Pinus* ssp. hybrid individuals were detected both by AFLPs and morphological traits (15% of all putative *Pe*), 163 putative hybrids by AFLPs (58% of all putative *Pe*) and 42 by morphological traits (15%) (Table 2). The morphological traits did not discover hybridization in 121 trees (74% of all detected hybrids using AFLPs). The current annual increment (CAI) did not significantly differ between the *Pe* trees and their hybrids.

**Table 2:** Mean Tanimoto distance and minimum number of hybrids per *Pinus engelmannii* stand.

Stand	Mean Tanimoto distance	AFLPs (No.) *Pe* x *Pinus* ssp.	AFLPs (No.) *Pe* x *Pco*	AFLPs (No.) *Pe* x *Pdu*	Morphological (No.)
ARC	0.40	1	1	1	0
CU	0.32	1	1	0	0
LM	0.53	1	1	1	0
NP	0.33	35	32	3	0
RC	0.48	23	19	7	0
MC	0.60	35	35	14	2 (*Pe* x *Par*, *Pe* x *Pmax*)
MCO	0.53	32	30	19	6 (*Pe* x *Par*)
R	0.62	35	35	8	34 (*Pe* x *Par*)

Mean Tanimoto distance and minimum number of hybrids detected by AFLPs (probability < 95% of affiliation of a putative *P*. *engelmannii* tree to the species, STUCTURE analysis with K = 2) and morphological traits per *P*. *engelmannii* seed stand. *Pe* = *Pinus engelmannii*, *Pco* = *P*. *arizonica* var. *cooperi*, *Pdu* = *P*. *durangensis*, *Par* = *P*. *arizonica* var. *arizonica*, *Pmax* = *P*. *maximinoi*, RC = *Pe* seed stand in Rancho Los Castro, MC = *Pe* seed stand in Mesa Cebollas, MCO = *Pe* seed stand in Mesa Coyotes, R = *Pe* seed stand in El Río, ARC = *Pe* seed stand in Adolfo Ruiz Cortinez, CU = *Pe* seed stand in Cumbres, LM = *Pe* seed stand in La Mesa, NP = *Pe* seed stand in Nueva Patria.

STRUCTURE analysis indicated that 55% of all *Pe* individuals had genetic introgression from *Pco*, and 19% from *Pdu* (Table 2). In addition, 3% of *Pco* in the MEX stand showed introgression from *Pe*, 100% of *Pco* from *Pe* in AB, 3% of *Pdu* from *Pe* in SE stand and 46% of *Pdu* from *Pe* in the VEN stand.

By the results of STRUCTURE analysis only eight of the putative *Pe* trees were identified as first-generation (*F*_*1*_) hybrids *Pe* x *Pco* (in each of these four stands: RC, R, MCO, and MC).

Statistically significant covariations (*C*) of the mean Tanimoto distance (*TD*) with hybrid frequency per *P*. *engelmannii* stand were found for the combination *TD* and frequency of *Pe* x *Pdu* (*C* = 0.86, *P* = 0.035), *Pe* x *Pco* x *Pdu* (*C* = 0.92, *P* = 0.020) and morphological traits (*C* = 0.99, *P* = 0.015).

## Discussion and Conclusions

The *Pinus engelmannii* trees share many AFLP fragments with *P*. *arizonica* var. *cooperi* and with *P*. *durangensis*, due to the relatively recent diversification of *Ponderosae* species and the very weak physical and reproductive barriers between them [34][35] [36][37][38][40] followed by introgression [30]. This interspecific gene transfer is maintained by: i) wind pollination, ii) weak reproductive isolating barriers, iii) longevity, iv) overlapping generations, v) large effective population sizes [30] and vi) the overlapping geographical extension (sympatric distribution) of these three *Ponderosae* species.

The STRUCTURE analysis showed that many individuals from several *P*. *engelmannii* seed stands were hybridized with *P*. *arizonica* and *P*. *durangensis*. Only eight first-generation (*F*_*1*_) hybrids were found and a relatively high frequency of introgressed individuals was observed, as [69] recently found for *Salix*. This appears to indicate that *F*_*1*_ hybrids are not stable and backcrosses are the rule, so the alternative hypothesis referred to lack of hybridization is discarded. These results are consistent with reports of interspecific hybridization between Apache pine and Arizona pine (e.g. [42] [43]). Morphological analysis confirmed hybridization in two stands (R and MC) in which *P*. *engelmannii* and *P*. *arizonica* var. *arizonica* grow together; however, other cases of hybridization evidenced by AFLPs were not morphologically detected, as found also by [69] for Siberian willows. Natural pairwise and triple hybrids have also been found in some Mexican *Quercus* [70]. The similar current annual increment (CAI) observed for hybrids and pure trees in the eight *P*. *engelmannii* stands under study suggests that hybrids and pure trees did not differ in terms of vigour or robustness [10]. Fitness advantages of pure vs introgressed and *F*_*1*_ individuals were not found and were not expected, because all the studied individuals were healthy, dominant and superior phenotypes (plus trees).

All the stands with putative *P*. *engelmannii* hybrids detected both by AFLPs as by morphological traits (R, MC, MCO) also showed the highest average values of the Tanimoto distance (Table 2), indicating: i) more heterogeneous genetic material [4], ii) higher genetic variation and therefore iii) the higher evolutionary potential in these stands [1] [71], and iv) that the morphological differentiation (hybrid/not hybrid) is strongly associated with the Tanimoto distance per stand.

The isolated El Rio stand included the highest degree of hybridization (morphologically almost 100%). This stand appears to represent a stable hybrid zone [8]. As the hybrids are not spatially or ecologically isolated from the parental species [9] [7], and the morphological traits are combinations of traits of those species (no novel characteristics were found), hybrid speciation is not plausible in the population.

In Mexican *Arbutus*, hybrids are common and backcrossing occasionally occurs, particularly in disturbed areas [72] [73]. Recent data [74] [75] indicate that hybridization can be accelerated by climate change. Global climate change is resulting in wide-scale habitat modification that will result in increasing opportunities for hybridization, in an analogous manner to other forms of anthropogenic disturbance [76]. The consequences of hybridization on the evolution of a species will depend, both on the relative fitness of hybrid offspring compared with offspring of pure species, as on the frequency of hybrid matings [76]. New research lines are necessary to explore how introgressive hybridization in *P*. *engelmannii* would influence its adaptation to environmental changes.

We conclude that natural pairwise hybrids are very common in the *P*. *engelmannii* stands under study and that these hybrids are sometimes not morphologically obvious. Considering that the AFLPs results are less biased in the estimation of hybrids, morphological traits are not a good proxy to estimate hybridization in this species.Thus, both morphological and molecular approaches are necessary to confirm the genetic identity of forest reproductive material. Molecular approaches should involve a combination of plastid data, nuclear and mitochondrial DNA sequences and crossing experiments. The genetic identity of forest reproductive material is essential for a complete understanding of tree species phylogeny, for developing effective breeding programs, and for seed quality and reforestation success [30] [44] [77].

## Supporting Information

S1 Dataset Data set used in this study. (XLSX)
